# Association between weight-adjusted-waist index and gynecologic cancers: a population-based study

**DOI:** 10.3389/fnut.2024.1449643

**Published:** 2024-09-13

**Authors:** Liyuan Fang, Xiaotong Li, Yuhang Fang, Yan Wang, Runxi Wang, Yi Xie, Ying Zhang

**Affiliations:** ^1^Department of Oncology, Guang’anmen Hospital of the Chinese Academy of Traditional Chinese Medicine, Beijing, China; ^2^Department of Surgery, Shanghai University of Traditional Chinese Medicine Affiliated Putuo Central Hospital, Shanghai, China; ^3^Beijing University of Chinese Medicine, Beijing, China

**Keywords:** weight-adjusted-waist index, obesity, non-linear, NHANES, gynecologic cancers

## Abstract

**Objective:**

This study aims to analyze the association between the weight-adjusted waist index (WWI) and the risk of gynecologic cancers, using data collected from the National Health and Nutrition Examination Survey (NHANES) from 2011 to 2016.

**Methods:**

We employed multiple logistic regression analysis to investigate the relationship between WWI and risk of gynecologic cancers. Subsequent subgroup analyses were performed on specific populations of interest. A restricted cubic spline model was used to explore potential non-linear relationships. Additionally, the effectiveness of WWI in predicting sarcopenia was assessed through Receiver Operating Characteristic (ROC) curve analysis. K-fold cross-validation was applied for model assessment.

**Results:**

Among the 4,144 participants, 98 self-reported having gynecologic cancers. In the fully adjusted model, WWI was significantly associated with the prevalence of gynecologic cancers (OR = 1.38, 95% CI: 1.02–1.88, *p* = 0.0344). Our findings indicate a linear positive association between WWI and the risk of gynecologic cancers. Subgroup analysis revealed that WWI had the strongest association with cervical cancer (OR = 1.46, 95% CI: 0.97–2.18, *p* = 0.0354) and endometrial cancer (OR = 1.39, 95% CI: 0.81–2.39, *p* = 0.0142). No significant association was found between WWI and the risk of ovarian cancer (OR = 1.16, 95% CI: 0.48–2.72, *p* = 0.5359). Restricted cubic spline analysis confirmed a linear relationship between WWI and the risk of cervical, endometrial, and ovarian cancers. ROC curve analysis demonstrated that WWI had superior predictive capability for gynecologic cancers.

**Conclusion:**

Elevated levels of WWI were significantly associated with an increased risk of gynecologic cancers in American women, displaying a stronger association than other obesity markers. Therefore, WWI may serve as a distinct and valuable biomarker for assessing the risk of gynecologic cancers, particularly cervical and endometrial cancers.

## 1 Introduction

Malignant tumors have become the second leading cause of death worldwide, creating a substantial health burden that includes diminished quality of life, strained healthcare systems, and significant economic impacts (1, 2). According to 2022 statistics from the International Agency for Research on Cancer, millions of women are diagnosed annually with ovarian, cervical, and endometrial cancers, posing severe threats to both their physical and mental health. In developed countries, the incidence rates of ovarian and cervical cancers have stabilized (3, 4); however, the incidence of endometrial cancer continues to rise (5). Studies indicate that BRCA1 mutations can increase the risk of ovarian cancer by up to 40% (6), while approximately 5–10% of endometrial cancer cases are driven by POLE mutations (7). Environmental factors, including exogenous hormones, radiation exposure, and heavy metal ions, are known to elevate the risk of gynecological cancers (8). In addition to genetic predispositions and environmental exposures, lifestyle factors such as smoking, obesity, and physical inactivity significantly increase the risk of developing gynecological malignancies (9). Globally, the prevalence of overweight and obesity is increasing, with obesity rates in adult women significantly surpassing those in men across all age groups (10). By 2030, obesity rates in certain regions are projected to exceed 50% (11) Traditional measures such as body mass index (BMI) and waist circumference (WC) have been used as indicators of obesity, but they have limitations, notably their inability to distinguish the distribution of adipose tissue (12).

The Weight-Adjusted Waist Index is a novel and straightforward anthropometric measure of obesity, gaining significant attention for its efficacy in assessing obesity and related health risks (13). Compared to BMI and WC, WWI differentiates more effectively between fat and muscle mass. It provides a more accurate measure of central obesity and the health implications of visceral fat (14). Studies have demonstrated that elevated WWI is associated with an increased risk of several conditions, including depression (15), hypertension (16), diabetic nephropathy (17), and secondary infertility (18). However, the relationship between WWI and the prevalence of gynecological cancers remains unexplored, highlighting the necessity for further investigation. This study utilizes data from NHANES collected between 2011 and 2016. Multivariate logistic regression and restricted cubic spline analyses are employed to investigate the association between WWI and gynecological cancers, providing robust and flexible modeling to capture complex relationships. Additionally, subgroup analyses are conducted to explore the association between WWI and gynecological cancers across different demographic and clinical subgroups, offering a comprehensive understanding of potential variations in risk.

## 2 Methods

### 2.1 Study population

**Figure 1:**
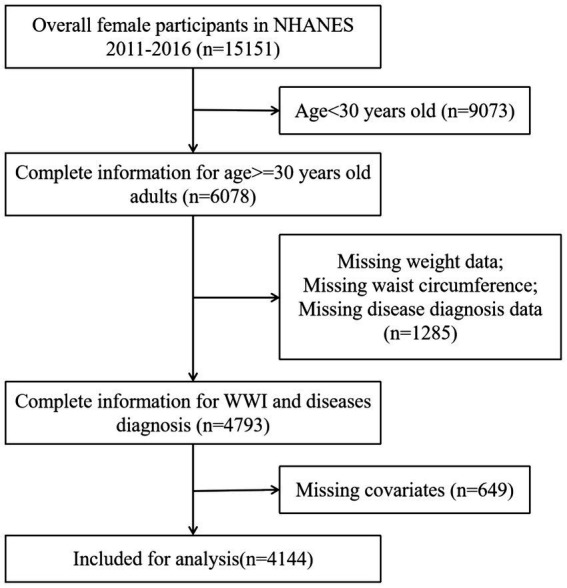
Flowchart of the sample selection from NHANES 2011–2016.

The National Health and Nutrition Examination Survey (NHANES), conducted by the National Center for Health Statistics (NCHS), employs a stratified multistage probability sampling method to obtain a representative sample of the civilian, non-institutionalized U.S. population. This methodology involves a structured selection process that includes counties, blocks, households, and individuals within those households. Since 1999, NHANES has conducted cross-sectional surveys, releasing new data every 2 years. To maintain the rigor and accuracy of the study, specific exclusion criteria were implemented, particularly the exclusion of participants under 30 years of age (19). This decision was informed by the widespread uptake of the HPV vaccine and heightened self-care awareness in this demographic, both of which have contributed to a lower incidence of gynecological tumors in individuals younger than 30. The detailed inclusion and exclusion criteria are presented in Figure 1. In summary, the study encompassed 4,144 participants. Among these participants, 98 self-reported a history of gynecologic cancers, including 12 with ovarian cancer, 56 with cervical cancer, and 30 with endometrial cancer. The NHANES protocol received approval from the NCHS Research Ethics Review Board, and informed consent was obtained from all participants prior to their inclusion in the study.

### 2.2 Calculation of WWI

The WWI was the primary exposure factor in this study. WWI was calculated for each participant by dividing the waist circumference (in centimeters) by the square root of the body weight (in kilograms). Anthropometric measurements were meticulously recorded by trained medical personnel and specialized recorders to ensure data accuracy. Body weight was measured using a digital scale. Participants were dressed in examination clothing, stood barefoot on the scale, held their arms close to their bodies, and fixed their gaze straight ahead, as previously outlined. WC was measured with a tape measure positioned at the intersection of the midaxillary line and a horizontal line just above the outermost upper point of the right kneecap.

### 2.3 Diagnosis of cancer

Data on cancer diagnoses were obtained from a structured questionnaire. Participants were asked if a doctor or other health professional had ever informed them of a cancer or malignancy diagnosis (MCQ-220). Participants who answered affirmatively were identified as cancer patients and were subsequently prompted to answer MCQ-230A. In MCQ-230A, code 15 indicates cervical cancer, code 28 indicates ovarian cancer, and code 38 indicates endometrial cancer.

### 2.4 Covariates

Based on previous research findings (20, 21), our study also accounted for additional variables, including age (years), the ratio of family income to poverty (PIR), race/ethnicity (Mexican American/Non-Hispanic White/Non-Hispanic Black/Other Race), education level (less than high school/high school graduate/more than high school), smoking status (ever smoked at least 100 cigarettes: yes/no), alcohol consumption (at least 12 drinks per year: yes/no), as well as platelet count, neutrophil count, lymphocyte count, and systemic inflammation index. PIR was characterized into three categories (22): low-income (PIR ≤ 1.3), middle-income (PIR > 1.3–3.5), and high-income (PIR > 3.5).

### 2.5 Statistical analysis

In accordance with the Centers for Disease Control and Prevention (CDC) recommendations on statistical analysis of complex survey data, all statistical analyses were carried out using the proper NHANES sampling weights and took into consideration intricate multistage cluster surveys. Continuous variables were presented as mean ± standard deviation, whereas categorical variables were represented as percentages. Differences between groups were evaluated using a weighted Student’s *t*-test (for continuous variables) or a weighted chi-square test (for categorical variables). Logistic regression was employed to examine the association between WWI and gynecological cancers, utilizing the adjusted odds ratio (OR) and the corresponding 95% confidence intervals (CI) to delineate the relationships. Following the Strengthening the Reporting of Observational Studies in Epidemiology (STROBE) guidelines (23), three multivariate regression models were constructed. In model 1, no covariates were adjusted. In model 2, age, race, ratio of family income to poverty, and education level were adjusted. Model 3 was adjusted for age, race, ratio of family income to poverty, education level, smoking status, alcohol status, BMI, platelet count, neutrophil count, lymphocyte count, and systemic inflammation index. Model 4, which included all variables from Model 3 except for BMI. To assess its robustness, the continuous variable WWI was categorized into tertiles for sensitivity analysis. We performed subgroup analyses and interactions for age, education level, smoking, alcohol status, and the ratio of family income to poverty in fully adjusted models. The predictive capacity of WWI for gynecological cancers was assessed using ROC curve analysis, obtaining area under the curve (AUC), sensitivity, and specificity values (24). In general, an AUC value of 0.5 indicates a lack of discrimination, while a range of 0.7–0.8 is deemed acceptable. As part of the model validation process, we employed *k*-fold cross-validation (*k* = 10) to rigorously assess the predictive performance of our model (25). In the fully adjusted model, we employed the restricted cubic spline (RCS) method to investigate the non-linear association between the WWI, serving as the exposure variable, and gynecological cancers outcome variable. Statistical analyses were conducted using R version 4.2.3 (http://www.R-project.org, The R Foundation). In the context of statistical analysis, a *p*-value less than 0.05 is considered statistically significant.

## 3 Results

### 3.1 Participant characteristics

This study encompassed a total of 4,144 participants, whose detailed characteristics are presented in Table 1. Compared to the normal group, patients diagnosed with gynecological tumors were generally older, had higher educational attainment, better economic status, a history of smoking, and greater waist circumferences.

**Table 1:** Baselines characteristics of participants.

Characteristic	Normal (*N* = 4,046)	Gynecologic cancers (*N* = 98)	*p*-value
Age (years)	49.28 + 11.63	52.59 + 11.12	0.0045
Race/ethnicity, *N*			<0.0001
Mexican American	592	17	
Other race	1,089	17	
Non-Hispanic White	1,410	56	
Non-Hispanic Black	955	8	
Education level, *N*			0.0318
Less than high school	330	13	
High school	476	17	
More than high school	3,240	68	
Family poverty ratio, *N*			0.0184
low-income	1,384	45	
middle-income	1,111	28	
high-income	1,551	25	
Smoking status, *N*			<0.0001
No	2,608	37	
Yes	1,438	61	
Alcohol status, *N*			0.5926
No	1,574	35	
Yes	2,472	63	
Weight (kg)	78.77 + 21.53	81.40 + 20.60	0.2149
WC (cm)	99.64 + 16.91	104.23 + 18.10	0.0143
BMI (kg/m^2^)	30.43 + 7.77	31.84 + 8.14	0.0916
Platelet count (1,000 cells/μL)	254.50 + 63.24	247.16 + 57.80	0.2184
neutrophils num (1,000 cell/μL)	0.52 + 0.18	0.53 + 0.18	0.709
Lymphocyte number (1,000 cells/μL)	2.23 + 0.73	2.34 + 0.74	0.1474
SII	527.21 + 306.15	536.18 + 274.40	0.7506

### 3.2 The association between WWI and gynecologic cancers

**Figure 2:**
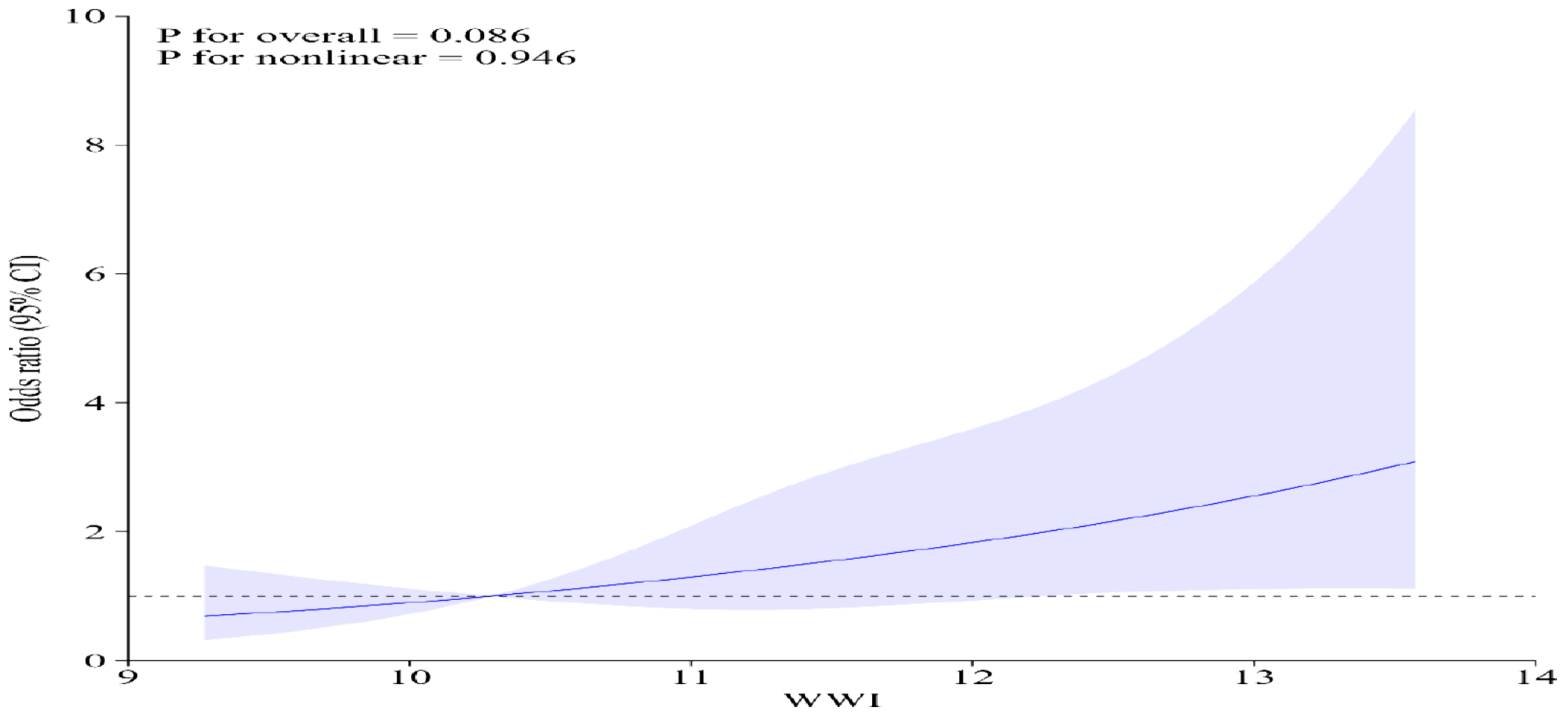
Restricted cubic spline analysis of WWI and gynecological tumors. Adjusted for age, race, ratio of family income to poverty, education level, smoking status, alcohol status, BMI, platelet count, neutrophil count, lymphocyte count, and systemic inflammation index.

Multivariate regression analysis demonstrated that, in the fully adjusted continuous model, each unit increase in WWI corresponded to a 38% higher risk of gynecological tumors (OR = 1.38 95%CI: 1.02–1.88 *p* = 0.0344). In the fully adjusted categorical model, the risk of gynecological tumors increased by 26 and 74% for the tertiles 2 and tertiles 3, respectively, compared to the tertile 1(*P* for trend = 0.0432) (Table 2). In Model 4, even when BMI is excluded, WWI still demonstrates a significant association with the risk of gynecologic cancers. Moreover, restricted cubic spline analysis indicated a positive linear trend (*P* for nonlinear = 0.946) (Figure 2).

**Table 2:** Associations between WWI and the risk of gynecological tumors.

Characteristic	Model 1 OR (95%CI), *p*-value	Model 2 OR (95%CI), *p*-value	Model 3 OR (95%CI), *p*-value	Model 4 OR (95%CI), *p*-value
WWI Index (Continuous)	1.63 (1.26–2.10) <0.0001	1.41 (1.01–1.85) 0.0148	1.38 (1.02–1.88) 0.0344	1.33 (1.01–1.79) 0.0435
Categories				
Tertile 1	1	1	1	1
Tertile 2	1.39 (0.8–2.48)	1.19 (0.67–2.14)	1.26 (0.70–2.32)	1.19 (0.67–2.15)
Tertile 3	2.33 (1.41–4.00)	1.76 (1.02–3.10)	1.74 (0.96–3.28)	1.65 (0.94–2.95)
*P* for trend	0.0009	0.0333	0.0432	0.0384

Insensitivity analysis, the weight-adjusted-waist index was converted from a continuous variable to a categorical variable (tertiles). 95% CI, 95% confidence interval; OR, odds ratio. Model 1: Covariates were not adjusted at all. Model 2: Adjusted for age, race, ratio of family income to poverty, and education level. Model 3: Adjusted for age, race, ratio of family income to poverty, education level, smoking status, alcohol status, BMI, platelet count, neutrophil count, lymphocyte count, and systemic inflammation index. Model 4: Adjusted for age, race, ratio of family income to poverty, education level, smoking status, alcohol status, platelet count, neutrophil count, lymphocyte count, and systemic inflammation index.

### 3.3 Subgroup analysis

**Figure 3:**
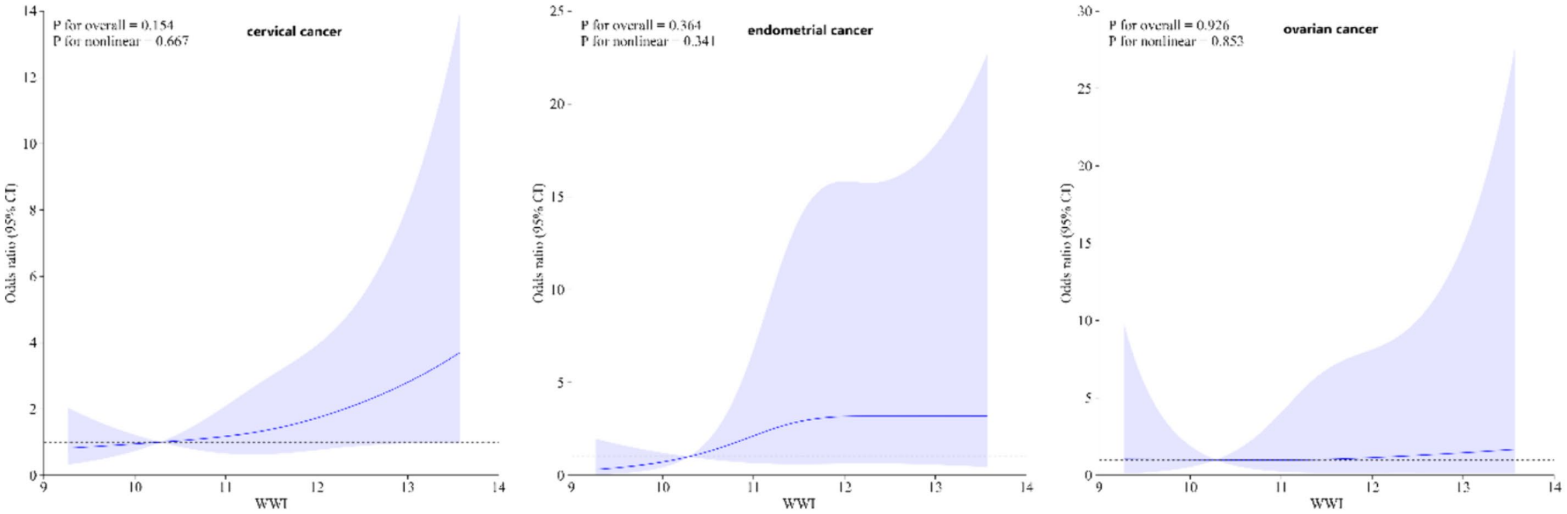
Restricted cubic spline analysis of WWI and the cervical, endometrial and ovarian cancer. Adjusted for age, race, ratio of family income to poverty, education level, smoking status, alcohol status, BMI, platelet count, neutrophil count, lymphocyte count, and systemic inflammation index.

**Figure 4:**
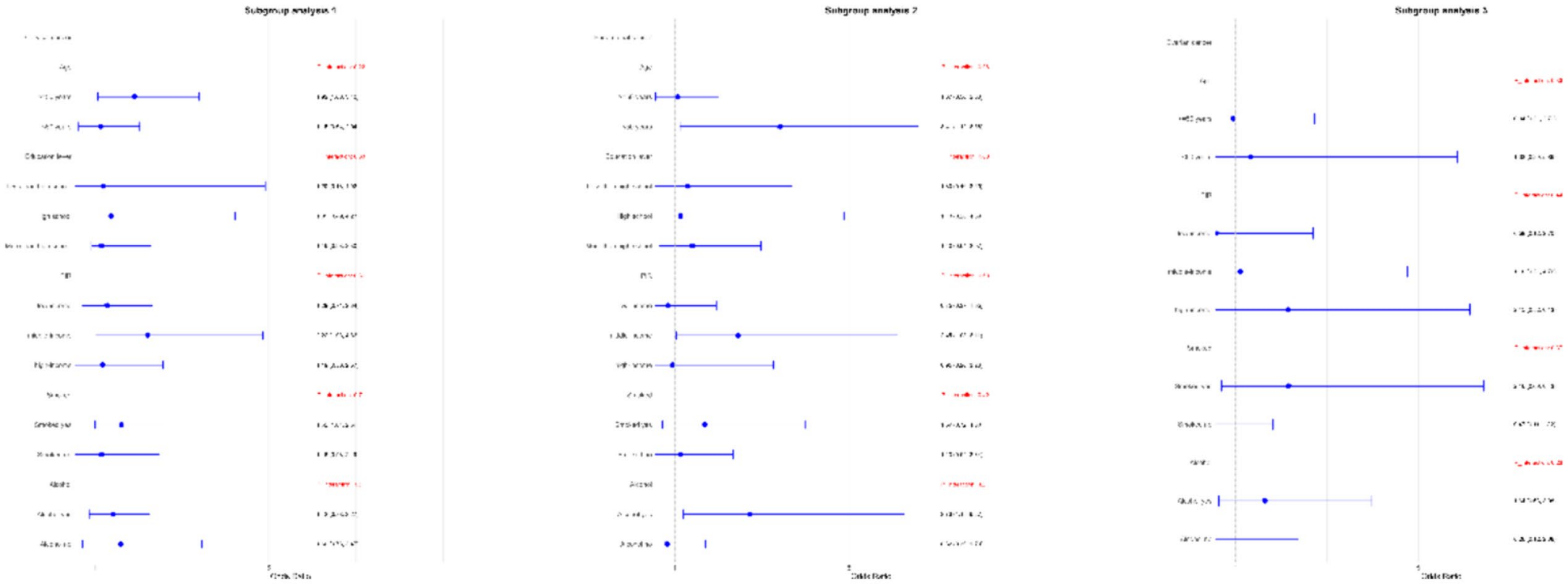
Subgroups analyses of the effect of WWI on cervical, endometrial and ovarian cancer.

**Figure 5:**
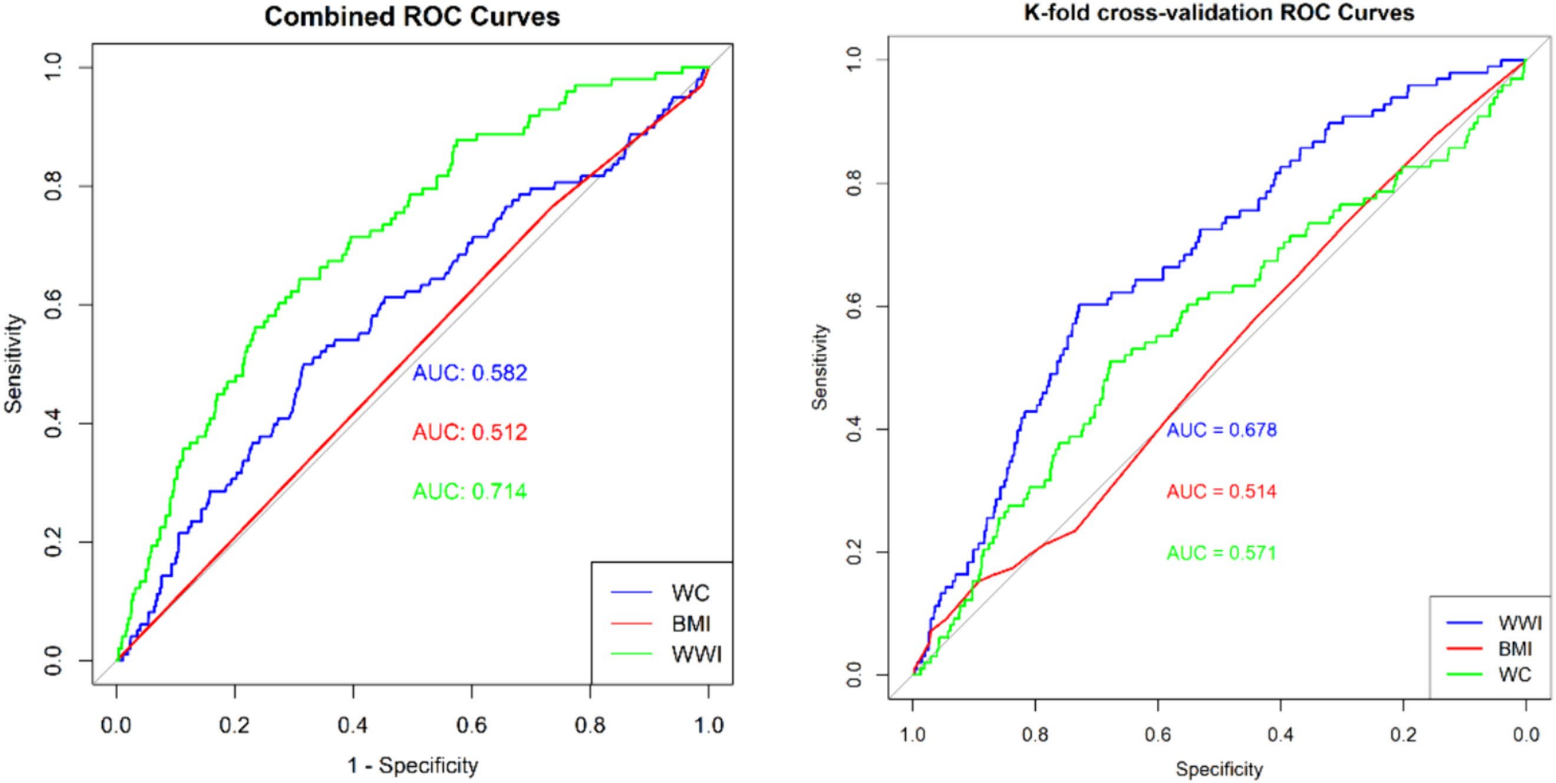
Area under the receiver operating characteristic curve of WWI, BMI and WC for predicting the gynecological tumors.

Subgroup analyses were performed to assess the relationship between WWI and the risk of ovarian, cervical, and endometrial cancers. The results indicated that, in the fully adjusted continuous model, each unit increase in WWI was associated with a 46% increase in the risk of cervical cancer (OR = 1.46 95%CI: 0.97–2.18 *p* = 0.0354) and a 39% increase in the risk of endometrial cancer (OR = 1.39 95%CI: 0.81–2.39 *p* = 0.0142). However, no statistically significant association was found between increased WWI and ovarian cancer risk (OR = 1.16 95%CI: 0.48–2.72 *p* = 0.5359) (Table 3). Restricted cubic spline analysis revealed a linear relationship between WWI and the risks of cervical, endometrial, and ovarian cancer (Figure 3). When WWI reaches 11, there is a marked increase in the risk of cervical and endometrial cancers, while the impact on ovarian cancer risk appears to be less pronounced. Although these curves demonstrate an upward trend, the associations observed do not achieve statistical significance. In addition, the subgroup analysis revealed that although variations in odds ratios were observed across different demographic and behavioral subgroups, the interactions between WWI and these factors—age, educational level, poverty status, smoking, and alcohol consumption—did not reach statistical significance. Specifically, while certain subgroups, such as individuals with middle income or those over 50 years old, exhibited higher odds ratios for specific cancers, the interaction *p*-values indicated no statistically significant differences (Figure 4). ROC curve analysis demonstrated that WWI had superior predictive capability compared to other obesity indicators, such as BMI and WC (Figure 5).

**Table 3:** Associations between WWI and the risk of cervical, endometrial and ovarian cancer.

Characteristic	Model 1 OR (95%CI), *p*-value	Model 2 OR (95%CI), *p*-value	Model 3 OR (95%CI), *p*-value	Model 4 OR (95%CI), *p*-value
WWI Index and cervical cancer	1.44 (1.03–2.00) 0.0245	1.36 (0.95–1.94) 0.0618	1.46 (0.97–2.18) 0.0354	1.32 (0.91–1.91) 0.0407
WWI Index and endometrial cancer	1.98 (1.26–3.10) 0.0031	1.46 (0.88–2.40) 0.0229	1.39 (0.81–2.39) 0.0142	1.36 (0.81–2.27) 0.0267
WWI Index and ovarian cancer	1.77 (0.86–3.59) 0.1161	1.51 (0.69–3.22) 0.2940	1.16 (0.48–2.72) 0.5359	1.20 (0.52–2.68) 0.4614

95% CI, 95% confidence interval; OR, odds ratio. Model 1: Covariates were not adjusted at all. Model 2: Adjusted for age, race, ratio of family income to poverty, and education level. Model 3: Adjusted for age, race, ratio of family income to poverty, education level, smoking status, alcohol status, BMI, platelet count, neutrophil count, lymphocyte count, and systemic inflammation index. Model 4: Adjusted for age, race, ratio of family income to poverty, education level, smoking status, alcohol status, platelet count, neutrophil count, lymphocyte count, and systemic inflammation index.

## 4 Discussion

In this cross-sectional study, we investigate the association between WWI and the incidence of gynecological cancers. Our findings indicate that an elevated WWI is linked to a high risk of gynecological malignancies, particularly cervical and endometrial cancers. The results of the subgroup analysis indicate that the association between WWI and gynecological cancers is not confounded by factors such as age, smoking, or alcohol consumption. This finding further underscores the importance of considering WWI as an independent risk factor for gynecological malignancies. Moreover, our research indicate that WWI may serve as a valuable independent predictor of gynecological cancer risk, even in the absence of BMI as a covariate.

Although obesity is widely recognized as a risk factor for cancer, the relationship between traditional measures of obesity, such as BMI and WC, and the risk of gynecological cancers remains unclear. Recent Mendelian randomization studies indicate that genetically predicted increases in BMI are linked to a higher risk of endometrial cancer (26). A cohort study involving 5 million individuals shows that for every 5 kg/m^2^ increase in BMI, the risks of endometrial and cervical cancers rise by 62 and 10%, respectively (27). Additionally, a large retrospective cohort study from Israel demonstrates an inverse association between higher adolescent BMI and the risk of cervical cancer in middle age (28). A meta-analysis reveals that being overweight or obese significantly increases the risk of ovarian cancer, with a 6% increase in risk for every 10 cm increase in WC (29, 30). Pathological analysis of ovarian cancer subtypes indicates that a higher BMI increases the risk of non-high-grade serous ovarian cancer but does not affect the risk of the more aggressive high-grade serous ovarian cancer (31). A cohort study finds no association between WC and BMI and the risk of epithelial ovarian cancer in premenopausal or postmenopausal women (32). Similarly, Mendelian randomization studies from Japanese and European populations show no association between higher BMI and ovarian cancer risk (33). BMI and WC are limited by their inability to distinguish between fat and muscle mass (34), often leading to the “obesity paradox.” Evidence suggests that during aging, the interplay between fat, muscle, and bone tissues results in changes in body composition, such as increased fat mass and decreased muscle and bone mass (35). A growing number of experts argue that BMI and WC are inadequate measures of obesity because they cannot differentiate between lean body mass and fat mass and are influenced by age, sex, and racial differences (36).

To the best of our knowledge, this is the first study to utilize the WWI to evaluate its relationship with the risk of gynecological cancers. Our findings demonstrate that a higher WWI is associated with an increased risk of gynecological malignancies. Compared to BMI and WC, WWI is a recently developed obesity assessment metric that accurately reflects the total body fat ratio and has been extensively studied across various fields. Liu et al. observed that WWI had a stronger association with depression compared to traditional indices like BMI and WC (15). Wang et al. found that in American adults over 60 years old, each unit increase in WWI was associated with a 32% increase in the prevalence of hypertension (16). This association persisted even after adjusting for age, sex, race, and adverse lifestyle factors. Xie et al. discovered that WWI was superior to BMI and WC in predicting severe abdominal aortic calcification (AAC). Higher WWI was significantly associated with severe AAC scores (37). Wen et al. found that among 3,526 participants, higher WWI values were linked to an increased incidence of infertility. Compared to other obesity metrics, including WC and BMI, WWI showed a stronger association with infertility risk (38).

The WWI is an essential metric for assessing abdominal fat, providing a more nuanced understanding of obesity. In gynecological cancers, adipose tissue primarily facilitates carcinogenesis through hormonal, inflammatory, and metabolic mechanisms (39). Adipose tissue, functioning as an active endocrine organ, secretes glucocorticoids and estrogens, among other hormones. Glucocorticoids facilitate the conversion of androgens to estrogens. Elevated estrogen levels, in turn, activate downstream mitotic signaling pathways, promoting cancer cell proliferation (40). Obesity often involves chronic low-grade inflammation, significantly altering metabolism and tissue homeostasis, and thereby leading to tumorigenesis (41). Chronic inflammation activates intracellular signaling pathways involving nuclear factor-κB, which regulates interleukin-6 (IL-6). IL-6, via its receptor and an intracellular cascade mediated by Janus kinase proteins, activates the signal transducer and activator of transcription 3. This activation leads to the expression of genes, including cyclins, which induce cell proliferation (42). Additionally, excessive accumulation of adipose tissue can result in metabolic disturbances, characterized by reduced glycolysis and oxidative phosphorylation. These changes impair the function of natural killer cells, CD8 T cells, and CD4 T cells, reducing their cytotoxicity and consequently increasing cancer risk (43, 44).

This study presents several strengths. Firstly, it uses NHANES data, ensuring the objectivity of the information. Secondly, we meticulously adjusted for confounding variables, which enhances the reliability of our findings and broadens their applicability. Thirdly, the study introduces and validates the WWI as a superior predictor of gynecological cancer risk compared to traditional obesity measures such as BMI and WC. In low-resource settings, where access to advanced diagnostic tools may be restricted, the WWI offers a valuable alternative for identifying individuals at high risk for gynecological cancers. The simplicity of WWI calculation, which relies on basic anthropometric measurements, makes it a cost-effective and easily implementable tool for early cancer detection. By incorporating WWI into routine screenings, healthcare providers can more effectively allocate resources and prioritize further diagnostic evaluations for those identified as high-risk.

However, this study has certain limitations. As a cross-sectional study, it cannot fully establish the relationship between WWI and the risk of gynecological cancers. Moreover, given that our data analysis is based on an American population, the generalizability of these findings to other populations remains uncertain. Future studies should validate the predictive power of WWI across cohorts with diverse ethnicities, regions, and age groups to enhance its generalizability and clinical utility. Furthermore, we acknowledge that the imbalance in sample size may affect the robustness of the statistical model. Future research should consider replicating our analyses in larger and more balanced cohorts to further validate the robustness of our findings. Self-reported data is a common practice in epidemiological studies, though its accuracy may be limited by recall bias or respondent misinterpretation. These limitations can result in inaccuracies in cancer diagnosis information, potentially impacting the reliability of the study’s findings.

## 5 Conclusion

The research indicates that elevated WWI levels are associated with an increased risk of gynecological cancers, particularly cervical and endometrial cancers. Compared to BMI and WC, WWI exhibits superior predictive capabilities and may serve as a valuable anthropometric indicator for assessing gynecological cancer risk.

## Data Availability

The original contributions presented in the study are included in the article/supplementary material, further inquiries can be directed to the corresponding author.
